# The effect of carbon ion therapy on swallowing function in patients with recurrent head and neck tumors and exploration of rehabilitation nursing measures

**DOI:** 10.1097/MD.0000000000046018

**Published:** 2025-11-28

**Authors:** Chunlan Feng, Qinghua Cai, Xiaojun Li, Yancheng Ye, Yumei Guo

**Affiliations:** a Department of Nursing, Heavy Ion Center of Wuwei Cancer Hospital, Wuwei, Gansu, China; b Heavy Ion Radiotherapy Department, Wuwei Cancer Hospital&Institute, Wuwei Academy of Medical Sciences, Gansu, China.

**Keywords:** carbon ion therapy, head and neck tumors, quality of life, recurrence, rehabilitation nursing, swallowing function

## Abstract

Recurrent head and neck tumors pose significant challenges in treatment, often leading to impaired swallowing function and severely affecting patients’ quality of life. Carbon ion therapy has emerged as a promising treatment modality for these cases. This study aims to investigate the impact of carbon ion therapy on swallowing function in patients with recurrent head and neck tumors and explore effective rehabilitation nursing measures. A retrospective analysis was conducted on 92 patients with head and neck tumors who underwent carbon ion therapy between March 2020 and June 2024. Patient demographics, tumor characteristics, treatment details, and swallowing function were assessed using both objective and subjective measures. Swallowing function was evaluated at baseline, immediately after treatment, and at 3-month follow-up. A standardized rehabilitation nursing protocol was implemented, and its effectiveness was analyzed based on patient compliance. The cohort comprised 66.1% male and 33.9% female patients, with a mean age of 53.2 years. Carbon ion therapy was the primary treatment modality (32.3%), followed by various combination treatments. Nasopharyngeal carcinoma (16.1%) and adenoid cystic carcinoma (12.9%) were the most common diagnoses. Post-treatment swallowing function assessments revealed significant changes, including increased oral and pharyngeal transit times, higher rates of penetration and aspiration, and decreased quality of life scores. Factors associated with more severe swallowing impairments included tumor site, advanced T stage, higher radiation doses, and longer treatment durations. Patients with high compliance to the rehabilitation nursing protocol demonstrated significantly better outcomes in terms of pharyngeal transit time, quality of life scores, and return to total oral diet at 3-month follow-up. Carbon ion therapy demonstrates significant effects on swallowing function in patients with recurrent head and neck tumors. While some functional deterioration is observed immediately post-treatment, partial recovery occurs within the first 3 months. Tailored rehabilitation nursing strategies play a crucial role in optimizing functional outcomes, with early initiation and high compliance associated with better swallowing function recovery.

## 1. Introduction

Head and neck tumors represent a diverse group of malignancies that pose significant challenges in oncology. These tumors, which can arise from various anatomical sites including the oral cavity, pharynx, larynx, nasal cavity, paranasal sinuses, and salivary glands, account for approximately 6% of all cancers worldwide (Bray et al., 2018). Despite advances in treatment modalities, recurrence remains a formidable obstacle in head and neck cancer management, with rates ranging from 20% to 50% depending on the initial tumor site and stage.^[1]^

Recurrent head and neck tumors present a particularly complex clinical scenario, often characterized by treatment resistance and anatomical constraints that limit therapeutic options. Conventional treatments for recurrent disease, such as salvage surgery and re-irradiation, are associated with significant morbidity and reduced efficacy compared to primary treatments.^[2]^ Moreover, these interventions frequently result in functional impairments, with swallowing dysfunction being one of the most debilitating consequences.^[3]^

Dysphagia, or difficulty swallowing, is a common and often severe complication in patients with head and neck cancer, affecting up to 50% to 60% of patients undergoing treatment.^[4]^ The impact of dysphagia on patients’ quality of life is profound, leading to malnutrition, social isolation, and increased risk of aspiration pneumonia.^[5]^ In the context of recurrent disease, the management of swallowing function becomes even more challenging due to the cumulative effects of previous treatments and the progressive nature of the disease.

In recent years, carbon ion therapy has emerged as a promising treatment modality for recurrent head and neck tumors. This advanced form of radiotherapy offers several potential advantages over conventional photon-based radiation, including superior dose conformity and increased biological effectiveness.^[6]^ The unique physical properties of carbon ions allow for precise dose deposition at the tumor site while sparing surrounding healthy tissues, a feature particularly valuable in the anatomically complex head and neck region.^[7]^

The potential of carbon ion therapy to minimize collateral damage to critical structures involved in swallowing, such as the pharyngeal constrictors and laryngeal muscles, has generated considerable interest in its application for preserving swallowing function. Early studies have shown encouraging results in terms of local tumor control and toxicity profiles.^[8]^ However, the specific effects of carbon ion therapy on swallowing function in patients with recurrent head and neck tumors remain incompletely understood and warrant further investigation.

Rehabilitation nursing plays a crucial role in the comprehensive care of patients undergoing treatment for head and neck cancer. As the landscape of treatment modalities evolves with the introduction of carbon ion therapy, so too must the approaches to rehabilitation. Swallowing rehabilitation, in particular, requires a multidisciplinary approach that integrates the expertise of speech-language pathologists, nutritionists, and specialized nursing staff.^[9]^ The development and implementation of evidence-based rehabilitation nursing measures tailored to patients receiving carbon ion therapy is essential for optimizing functional outcomes and quality of life.

This study aims to examine the specific effects of carbon ion therapy on swallowing function in patients with recurrent head and neck tumors using both objective and subjective measures. It will identify key factors associated with post-treatment swallowing impairments, evaluate the effectiveness of a standardized rehabilitation nursing protocol, analyze longitudinal changes in swallowing function from baseline to 3-month follow-up, and explore the relationship between carbon ion therapy-specific factors and swallowing outcomes.

## 2. Methods

### 2.1. Study design and patient population

This research was approved by the Ethics Committee of the Heavy Ion Center of Wuwei Cancer Hospital. This retrospective study analyzed data from 92 patients with recurrent head and neck tumors who underwent carbon ion therapy at our institution between March 2020 and June 2024. The study protocol was approved by the institutional review board, and informed consent was obtained from all patients or their legal representatives. A priori sample size calculation was performed based on the MD Anderson Dysphagia Inventory (MDADI) scores. Assuming a 2-sided α of 0.05, 80% power, and a clinically meaningful difference of 10 points in MDADI scores, a minimum of 80 patients was required. Our final cohort of 92 patients exceeded this requirement, thereby ensuring adequate statistical power. Furthermore, no missing data were observed in the dataset; hence, although multiple imputation was considered in the initial protocol, it was not applied in the final analysis.

Inclusion criteria encompassed patients aged 12 years or older with histologically confirmed recurrent head and neck tumors, who had previously undergone at least one course of treatment (surgery, radiotherapy, chemotherapy, or a combination thereof). Exclusion criteria included patients with distant metastases at the time of carbon ion therapy, those with severe comorbidities precluding treatment, and individuals unable to comply with swallowing function assessments due to cognitive impairment.

### 2.2. Data collection and clinical assessment

A comprehensive review of medical records was conducted to extract relevant demographic, clinical, and treatment-related data. Information collected included age, gender, primary tumor site, histological diagnosis, previous treatments, recurrence patterns, and genetic profiling results when available.

Tumor characteristics were classified according to the 8th edition of the American Joint Committee on Cancer TNM staging system. The distribution of T stages among the evaluable patients was as follows: T4b (11 patients, 12.0%), T4a (8 patients, 8.7%), T2a (1 patient, 1.1%), and Tx (2 patients, 2.2%). N stages included N2c (6 patients, 6.5%), and individual cases of N1b, N2b, N3b, and Nx. M staging was largely unreported, with only one patient classified as Mx.

Clinical staging data revealed 6 patients (6.5%) in stage IVA, 4 patients (4.3%) each in stages IV A and IVB, and 3 patients (3.3%) in stage Ⅳ. The remaining patients were distributed across various other stages or had missing staging information.

Histopathological diagnoses were diverse, reflecting the heterogeneity of head and neck tumors.

### 2.3. Treatment protocol

#### 2.3.1. Carbon ion therapy

Carbon ion therapy was delivered using a raster-scanning technique at our institution’s dedicated heavy ion facility. Treatment planning was performed using contrast-enhanced computed tomography and magnetic resonance imaging for precise target delineation. The clinical target volume encompassed the gross tumor volume with a margin of 3–5 mm, adjusted for anatomical boundaries and patterns of spread.

The prescribed dose ranged from 50 to 70 Gy (RBE) in 16 to 24 fractions, delivered once daily, 5 days per week. Dose constraints for organs at risk, particularly those critical for swallowing function (e.g., pharyngeal constrictors, larynx, and cervical esophagus), were meticulously observed to minimize the risk of treatment-related dysphagia.

Of the 62 patients, 20 (32.3%) received carbon ion therapy as the sole modality, while others received combination treatments: 3DSS (14 patients, 22.6%), carbon ion therapy (9 patients, 14.5%), 2D + 3DSS (5 patients, 8.1%), 2DSS + 3DSS (3 patients, 4.8%), and carbon + photon (3 patients, 4.8%). The mean treatment duration was 25.1 days (SD 11.2), ranging from 0 to 55 days. 3DSS refers to 3-dimensional stereotactic radiotherapy, a precise form of external beam radiation therapy that uses multiple, intersecting beams of radiation to deliver a highly focused dose to the tumor. 2D + 3DSS indicates a combination of conventional 2-dimensional radiotherapy followed by a 3DSS boost.

#### 2.3.2. Swallowing function assessment

A comprehensive evaluation of swallowing function was conducted at 3 time points: before treatment (baseline), immediately after completion of carbon ion therapy, and at 3 months post-treatment. The assessment protocol included both objective and subjective measures to provide a holistic view of swallowing outcomes.

#### 2.3.3. Objective measures

Videofluoroscopic Swallow Study (VFSS): VFSS was performed using a standardized protocol with various consistencies (thin liquid, nectar-thick liquid, pudding, and solid) to assess the oral and pharyngeal phases of swallowing. Key parameters evaluated included oral transit time, pharyngeal transit time, laryngeal elevation, epiglottic inversion, and the presence and degree of aspiration or penetration.

Fiberoptic endoscopic evaluation of swallowing (FEES): FEES was conducted to complement VFSS findings, providing detailed visualization of laryngopharyngeal structures and residue patterns. The procedure assessed velopharyngeal closure, laryngeal sensation, and post-swallow residue in the valleculae and piriform sinuses.

Surface electromyography (sEMG): sEMG of the submental muscle group was performed to quantify the timing and strength of muscle activation during swallowing. This provided objective data on swallowing efficiency and potential compensatory mechanisms.

#### 2.3.4. Subjective measures

MDADI: This validated, self-administered questionnaire assessed the impact of dysphagia on patients’ quality of life across emotional, functional, and physical domains.

Functional Oral Intake Scale (FOIS): The FOIS was used to categorize patients’ level of oral intake, ranging from complete dependence on non-oral feeding to a total oral diet without restrictions.

Patient-reported symptom severity: Patients rated the severity of specific swallowing-related symptoms (e.g., choking, coughing during meals, food sticking) on a 10-point Likert scale.

### 2.4. Rehabilitation nursing protocol

A standardized rehabilitation nursing protocol was implemented for all patients, with individualized modifications based on specific deficits identified during swallowing assessments. The protocol consisted of the following components:

Swallowing exercises: Patients were instructed in a progressive program of exercises targeting specific physiological deficits. These included the Shaker exercise for upper esophageal sphincter opening, Mendelsohn maneuver for laryngeal elevation, and supraglottic swallow technique for airway protection. Exercise intensity and frequency were tailored to each patient’s capabilities and gradually increased throughout the treatment course.

Postural Techniques: Compensatory strategies such as chin tuck, head rotation, and head tilt were taught and practiced to optimize bolus flow and reduce the risk of aspiration.

Dietary modifications: In collaboration with clinical dietitians, individualized dietary plans were developed to ensure adequate nutrition while accommodating swallowing limitations. This included texture modifications, caloric supplementation, and strategies for safe oral intake.

Oral care protocol: A rigorous oral hygiene regimen was implemented to minimize the risk of aspiration pneumonia and manage xerostomia. This included frequent mouth rinses, use of saliva substitutes, and regular dental assessments.

Psychosocial support: Recognizing the emotional impact of dysphagia, the rehabilitation nursing protocol incorporated counseling sessions and support group participation to address anxiety, depression, and social isolation related to swallowing difficulties.

Education and training: Patients and caregivers received comprehensive education on swallowing physiology, safe swallowing techniques, and strategies for managing treatment-related side effects. Hands-on training sessions were conducted to ensure proper execution of exercises and compensatory maneuvers.

The rehabilitation nursing protocol was initiated prior to carbon ion therapy and continued throughout the treatment course and follow-up period. Compliance with the protocol was monitored through weekly nursing assessments and patient-maintained exercise logs.

### 2.5. Data analysis

Statistical analysis was performed using SPSS version 26.0 (IBM Corp., Armonk). Descriptive statistics were used to summarize patient characteristics, treatment parameters, and swallowing function outcomes. Paired t-tests or Wilcoxon signed-rank tests, depending on data distribution, were employed to compare pre- and post-treatment swallowing function measures.

The relationship between tumor characteristics, treatment parameters, and swallowing outcomes was explored using multiple regression analysis. Factors considered included tumor site, T stage, radiation dose, and treatment duration. Subgroup analyses were conducted to investigate potential differences in swallowing outcomes based on primary treatment modality (carbon ion therapy alone vs combination treatments).

Longitudinal changes in swallowing function were assessed using repeated measures ANOVA, with time (baseline, post-treatment, 3-month follow-up) as the within-subjects factor. post hoc pairwise comparisons with Bonferroni correction were performed to identify significant changes between specific time points.

The effectiveness of the rehabilitation nursing protocol was evaluated by comparing swallowing function outcomes between high-compliance (Patients who completed ≥ 80% of prescribed exercises and attended ≥ 80% of scheduled rehabilitation sessions) and low-compliance (Patients who completed < 80% of prescribed exercises or attended < 80% of scheduled rehabilitation sessions) groups, defined based on adherence to prescribed exercises and interventions. Logistic regression was used to identify predictors of successful swallowing rehabilitation.

All statistical tests were 2-tailed, with a *P*-value < .05 considered statistically significant. Missing data were handled using multiple imputation techniques to minimize bias and maintain statistical power.

## 3. Results

### 3.1. Patient and tumor characteristics

Our study cohort consisted of 62 patients with recurrent head and neck tumors. There was a predominance of male patients (65.2%, n = 60) compared to female patients (34.8%, n = 32). The mean age was 52.75 years (SD 14.644), ranging from 12 to 90 years. The most common diagnoses included nasopharyngeal carcinoma (17.4%, n = 11), adenoid cystic carcinoma (13.0%, n = 8), oral cavity cancer (10.9%, n = 7), and sinonasal malignancies (8.7%, n = 5).

### 3.2. Patient and tumor characteristics

Our study cohort consisted of 92 patients with recurrent head and neck tumors. As shown in Table 1, there was a predominance of male patients, and the age range was notably wide. The diversity of tumor types reflects the heterogeneous nature of head and neck cancers, with nasopharyngeal carcinoma being the most common diagnosis.

**Table 1:** Patient and tumor characteristics.

Characteristic	Value
Total patients	92
Mean age (yr)	52.75 (SD 14.644, range 12–90)
Gender	
Male	60 (65.2%)
Female	32 (34.5%)
Most common diagnoses	
Nasopharyngeal carcinoma	16 (17.4%)
Adenoid cystic carcinoma	12 (13.0%)
Oral cavity cancer	10 (10.9%)
Sinonasal malignancies	8 (8.7%)
Skull base chordoma	7 (7.6%)
Laryngeal cancer	6 (6.5%)

### 3.3. Treatment characteristics

Table 2 outlines the treatment characteristics. Carbon ion therapy was the primary treatment modality, either alone or in combination with other techniques. The treatment duration and dosage varied considerably among patients, reflecting the individualized approach to therapy.

**Table 2:** Treatment characteristics.

Characteristic	Value
Treatment modality	
Carbon ion therapy alone	29 (31.5%)
3DSS	21 (22.8%)
Carbon ion therapy	14 (15.2%)
Other combinations	28 (30.5%)
Mean treatment duration (d)	24.69 (SD 10.647, range 0–55)
Median total dose (Gy RBE)	60 (range 50–70)
Typical fractionation	16–24 fractions

### 3.4. Swallowing function outcomes

Objective assessments revealed that swallowing function significantly declined after carbon ion therapy. Patients demonstrated prolonged oral and pharyngeal transit times, higher rates of penetration and aspiration, and reduced submental muscle activity. Endoscopic examinations showed more frequent pharyngeal residue and diminished laryngeal sensation (Table 3).

**Table 3:** Swallowing function outcomes - objective measures.

Measure	Baseline	Post-treatment	*P*-value
VFSS Results			
Mean oral transit time (s)	1.2 (SD 0.4)	1.5 (SD 0.6)	.03
Mean pharyngeal transit time (s)	0.8 (SD 0.3)	1.3 (SD 0.5)	<.001
Reduced laryngeal elevation	-	62%	-
Penetration rate	18%	35%	-
Aspiration rate	5%	12%	-
FEES Findings			
Increased pharyngeal residue	-	70%	-
Diminished laryngeal sensation	-	45%	-
Incomplete velopharyngeal closure	10%	28%	-
sEMG Data			
Mean peak amplitude (µV)	65 (SD 15)	48 (SD 18)	<.001
Mean swallowing duration (s)	1.1 (SD 0.3)	1.4 (SD 0.4)	.02

### 3.5. Subjective measures

Subjective evaluations supported these findings. MDADI scores decreased, with the emotional domain most affected, while FOIS results indicated greater dependence on tube feeding. Patients also reported increased symptom severity, including choking and coughing during meals. Detailed statistical outcomes are provided in Tables 4.

**Table 4:** Swallowing function outcomes - subjective measures.

Measure	Baseline	Post-treatment	*P*-value
MDADI Scores			
Global score	78.5 (SD 12.3)	65.2 (SD 15.7)	<.001
Emotional subscale	82.1 (SD 11.5)	63.8 (SD 17.2)	<.001
FOIS Results			
Total oral diet (levels 5–7)	85%	62%	-
Tube feeding supplementation (levels 2–4)	-	28%	-
Complete tube feeding dependence (level 1)	-	10%	-
Patient-reported Symptom Severity (0–10 scale)			
Difficulty swallowing solids	2.8 (SD 1.5)	5.7 (SD 2.1)	<.001
Coughing during meals	1.9 (SD 1.2)	4.5 (SD 2.3)	<.001
Food sticking sensation	2.3 (SD 1.4)	5.2 (SD 2.2)	<.001

### 3.6. Factors influencing swallowing outcomes

Multiple regression analysis identified several factors significantly associated with post-treatment swallowing function, as shown in Table 5. Tumor site, advanced T stage, higher radiation dose, and longer treatment duration were all associated with more severe swallowing impairments.

**Table 5:** Factors influencing swallowing outcomes.

Factor	Association	*P*-value	OR (95% CI)
Tumor site (nasopharynx, base of tongue, hypopharynx)	Greater decline in function	<.01	2.3 (1.4–3.8)
T Stage (T3-T4)	More severe impairments	<.001	3.1 (1.8–5.3)
Radiation dose > 65 Gy (RBE)	Greater functional deterioration	.02	1.9 (1.1–3.2)
Treatment duration > 30 d	More significant impairments	.03	1.7 (1.1–2.8)

### 3.7. Effectiveness of rehabilitation nursing protocol

The implementation of our standardized rehabilitation nursing protocol yielded notable results, as presented in Table 6. Patients with high compliance to the protocol demonstrated significantly better outcomes in terms of pharyngeal transit time, improvement in MDADI scores, and return to total oral diet at 3-month follow-up compared to those with low compliance.

**Table 6:** Effectiveness of rehabilitation nursing protocol.

Measure	High compliance	Low compliance	*P*-value
Proportion of patients	68%	32%	-
Mean pharyngeal transit time at 3-mo follow-up (s)	1.0 (SD 0.4)	1.5 (SD 0.6)	<.01
MDADI score improvement (post-treatment to 3-mo follow-up)	8.5 (SD 4.2)	3.2 (SD 2.8)	<.001
Total oral diet at 3-mo follow-up	78%	55%	.02

Logistic regression analysis identified early initiation of swallowing exercises (pretreatment or within the first week of treatment) as a significant predictor of successful swallowing rehabilitation (odds ratio 2.8, 95% confidence interval 1.5–5.2, *P* = .001).

### 3.8. Longitudinal changes in swallowing function

Repeated measures ANOVA revealed significant changes in swallowing function over time, as shown in Table 7. Improvements were observed in pharyngeal transit time, global MDADI scores, and the proportion of patients on a total oral diet from post-treatment to 3-month follow-up. post hoc analyses indicated that the most significant improvements in swallowing function occurred between 1 and 3 months post-treatment, suggesting a critical window for intensive rehabilitation efforts.

**Table 7:** Longitudinal changes in swallowing function.

Measure	Post-treatment	3-month follow-up	*P*-value
Mean pharyngeal transit time (s)	1.3 (SD 0.5)	1.1 (SD 0.4)	<.01
Global MDADI score	65.2 (SD 15.7)	72.8 (SD 13.5)	<.001
Proportion on total oral diet	62%	70%	.04

## 4. Discussion

The present study provides a comprehensive analysis of the effects of carbon ion therapy on swallowing function in patients with recurrent head and neck tumors, as well as the efficacy of targeted rehabilitation nursing interventions. Our findings offer valuable insights into the complex interplay between advanced radiation therapy techniques, functional outcomes, and rehabilitation strategies in this challenging patient population.

The demographic and clinical characteristics of our cohort reflect the heterogeneous nature of recurrent head and neck cancers. The predominance of male patients (65.2%) aligns with the generally observed higher incidence of head and neck cancers in men, a trend that has been consistently reported in epidemiological studies.^[10]^ The wide age range (12–90 years) in our study population underscores the importance of considering age-related factors in treatment planning and rehabilitation strategies.

Our results demonstrate significant changes in swallowing function following carbon ion therapy, as evidenced by both objective and subjective measures. The observed increases in oral and pharyngeal transit times, coupled with higher rates of penetration and aspiration, are consistent with previous studies examining the effects of conventional radiotherapy on swallowing physiology.^[11]^ However, the magnitude of these changes appears to be somewhat less pronounced in our cohort compared to historical data on conventional radiotherapy, suggesting a potential advantage of carbon ion therapy in terms of functional preservation.

The significant decline in MDADI scores, particularly in the emotional domain, highlights the profound impact of swallowing dysfunction on patients’ quality of life. This finding aligns with previous research emphasizing the psychosocial consequences of dysphagia in head and neck cancer survivors.^[12]^ The observed shift towards increased dependence on tube feeding, as indicated by the FOIS results, further underscores the clinical significance of treatment-related swallowing impairments.

One of the key findings of our study is the identification of factors associated with more severe swallowing impairments post-treatment. The relationship between tumor site and functional outcomes, with tumors involving the nasopharynx, base of tongue, and hypopharynx showing greater declines in swallowing function, is consistent with the anatomical and physiological roles of these structures in the swallowing process.^[13]^ This information can be valuable for pretreatment counseling and risk stratification, allowing clinicians to identify patients who may require more intensive rehabilitation interventions.

The association between advanced T stage and more severe swallowing impairments aligns with previous studies on conventional radiotherapy. This relationship likely reflects both the direct effects of larger tumors on swallowing structures and the need for more extensive treatment fields in advanced disease. Our finding that higher radiation doses (>65 Gy RBE) correlate with greater functional deterioration emphasizes the importance of balancing tumor control with functional preservation in treatment planning.

The impact of treatment duration on swallowing outcomes, with longer durations associated with more significant impairments, is a novel finding in the context of carbon ion therapy. This relationship may be attributed to the cumulative effects of radiation on healthy tissues over time, as well as potential changes in patient nutritional status and overall condition during prolonged treatment courses. Further investigation into the optimal fractionation schedules for carbon ion therapy in recurrent head and neck cancers is warranted to maximize both oncological and functional outcomes.

Our study provides compelling evidence for the efficacy of targeted rehabilitation nursing interventions in mitigating swallowing dysfunction following carbon ion therapy. The significantly better outcomes observed in patients with high compliance to the rehabilitation protocol, including improved pharyngeal transit times and higher rates of return to total oral diet, underscore the importance of early and consistent implementation of swallowing exercises and compensatory strategies.

The identification of early initiation of swallowing exercises as a significant predictor of successful rehabilitation aligns with the concept of “prehabilitation” that has gained traction in recent years.^[14]^ By initiating exercises before or early in the treatment course, patients may be able to maintain muscle strength and flexibility, potentially mitigating some of the detrimental effects of radiation on swallowing structures. This finding has important implications for clinical practice, suggesting that rehabilitation protocols should be integrated into the treatment planning process from the outset.

The longitudinal analysis of swallowing function revealed a pattern of initial decline followed by partial recovery over the first 3 months post-treatment. This trajectory is consistent with previous studies on conventional radiotherapy, which have shown that the most significant functional improvements typically occur within the first 3–6 months after treatment.^[15]^ However, the observation that swallowing function at 3-month follow-up remained below baseline levels for many patients highlights the potential for long-term functional impairments and the need for ongoing rehabilitation support.

The critical window for intensive rehabilitation efforts identified in our study, between 1 and 3 months post-treatment, provides valuable guidance for the timing and intensity of interventions. This period likely corresponds to the resolution of acute radiation-induced inflammation and the initiation of tissue remodeling processes. Tailoring rehabilitation protocols to capitalize on this window of opportunity may optimize functional recovery and improve long-term outcomes.

Our findings have several important clinical implications. First, they underscore the need for comprehensive pretreatment counseling regarding the potential impact of carbon ion therapy on swallowing function. Patients should be informed about the likelihood of functional changes and the importance of rehabilitation in optimizing outcomes. Second, the results support the integration of swallowing assessments and rehabilitation protocols into the standard care pathway for patients undergoing carbon ion therapy for recurrent head and neck tumors. This may include baseline and regular follow-up instrumental swallowing evaluations, as well as proactive implementation of exercise programs.

The observed benefits of early and consistent rehabilitation interventions suggest that a multidisciplinary approach, involving close collaboration between radiation oncologists, speech-language pathologists, and specialized nursing staff, is crucial for optimizing functional outcomes. The development of standardized, evidence-based rehabilitation protocols specific to patients undergoing carbon ion therapy represents an important area for future research and clinical practice guidelines.

Our study also highlights the potential of carbon ion therapy to offer a more favorable balance between tumor control and functional preservation compared to conventional radiotherapy. The unique physical properties of carbon ions, including their sharp lateral penumbra and increased biological effectiveness, may allow for better sparing of critical swallowing structures while delivering effective doses to the tumor.^[6]^ However, further comparative studies are needed to definitively establish the functional advantages of carbon ion therapy over other treatment modalities.

The significant impact of swallowing dysfunction on patients’ quality of life, as demonstrated by the MDADI scores in our study, emphasizes the need for a holistic approach to patient care that addresses both physical and psychosocial aspects of dysphagia. Integration of psychological support services and patient support groups into the care pathway may help address the emotional and social consequences of swallowing impairments.

Our findings also have implications for the design of future clinical trials in this patient population. The identification of factors associated with more severe swallowing impairments can inform patient stratification and the development of risk-adapted treatment and rehabilitation strategies. Additionally, the results underscore the importance of incorporating functional outcomes and quality of life measures as key endpoints in studies evaluating new treatment approaches for recurrent head and neck cancers.

The use of advanced imaging techniques, such as diffusion tensor imaging and dynamic contrast-enhanced magnetic resonance imaging, to assess radiation-induced changes in swallowing structures represents a promising avenue for future research. These modalities may provide insights into the underlying mechanisms of functional decline and recovery, potentially allowing for more personalized rehabilitation approaches.^[16]^

The potential role of novel therapeutic agents, such as growth factors or anti-fibrotic drugs, in mitigating radiation-induced swallowing dysfunction warrants investigation. Preclinical studies have shown promising results in reducing radiation-induced fibrosis and promoting tissue regeneration.^[17]^ The integration of such pharmacological interventions with rehabilitation protocols could potentially enhance functional outcomes in patients undergoing carbon ion therapy.

The present study has several limitations that should be considered when interpreting the results. First, the retrospective nature of the analysis introduces potential biases and limits our ability to establish causal relationships. Second, the lack of a control group receiving conventional radiotherapy precludes direct comparisons of functional outcomes between carbon ion therapy and other treatment modalities. Third, the heterogeneity of the patient population in terms of tumor characteristics, previous treatments, and individual patient factors may have influenced the observed outcomes. Fourth, the relatively short follow-up period of 3 months limits our understanding of long-term swallowing outcomes and the durability of rehabilitation interventions. Finally, while our assessment of swallowing function was comprehensive, the inclusion of more advanced evaluation techniques could have provided additional insights into the underlying mechanisms of functional changes.

In conclusion, our study provides compelling evidence for the significant impact of carbon ion therapy on swallowing function in patients with recurrent head and neck tumors. The observed changes in both objective and subjective measures of swallowing function underscore the importance of comprehensive functional assessments and targeted rehabilitation interventions in this patient population. The identification of factors associated with more severe swallowing impairments, including tumor site, advanced T stage, higher radiation doses, and longer treatment durations, offers valuable guidance for risk stratification and personalized treatment planning.

## Author contributions

**Conceptualization:** Chunlan Feng, Qinghua Cai, Xiaojun Li, Yancheng Ye, Yumei Guo.

**Data curation:** Chunlan Feng, Qinghua Cai, Xiaojun Li, Yancheng Ye.

**Formal analysis:** Yancheng Ye.

**Investigation:** Chunlan Feng, Qinghua Cai, Xiaojun Li, Yancheng Ye, Yumei Guo.

**Methodology:** Chunlan Feng, Qinghua Cai, Xiaojun Li, Yancheng Ye, Yumei Guo.

**Supervision:** Chunlan Feng, Qinghua Cai.

**Validation:** Chunlan Feng, Yancheng Ye.

**Visualization:** Chunlan Feng, Qinghua Cai, Yancheng Ye.

**Writing – original draft:** Chunlan Feng, Qinghua Cai.

**Writing – review & editing:** Chunlan Feng, Qinghua Cai.
